# Computer-navigated versus conventional total knee arthroplasty: no difference in implant survival at 15-year follow-up

**DOI:** 10.1186/s13018-026-06761-z

**Published:** 2026-03-11

**Authors:** Giovanni Longo, Giacomo Pacchiarotti, Carmela Pizzigallo, Stefano Gumina, Alessandro Todesca

**Affiliations:** 1 Department of Anatomy, Histology, Legal Medicine, and Orthopaedics, Sapienza University of Rome, Rome, Italy; 2 Casa di Cura Villa Betania, Giomi Group, Rome, Italy; 3 Istituto Chirurgico Ortopedico Traumatologico (ICOT), Latina, Italy

**Keywords:** Total knee arthroplasty, Computer-assisted surgery, Navigation, Implant survival, Long-term follow-up

## Abstract

**Introduction:**

The introduction of computer-assisted navigation (CAS) in total knee arthroplasty (TKA) aimed to improve prosthetic alignment and potentially enhance long-term implant survival. However, the actual clinical benefit beyond 10 years remains debated.

**Materials and methods:**

Retrospective comparative study with up to 15–16 years of follow-up, including patients undergoing TKA using either a conventional (CONV) or computer-navigated (NAV) technique. The primary outcome was implant survival (absence of revision for any cause). Secondary outcomes were functional scores: Knee Society Score (KSS) and Western Ontario and McMaster Universities Osteoarthritis Index (WOMAC).

**Results:**

Fifteen-year implant survival was 70.5% (CONV) vs. 73.4% (NAV) (*p* > 0.05). No statistically significant differences were found in functional outcomes. Mortality unrelated to the prosthesis was high in both groups.

**Conclusions:**

At 15 years, the navigated technique did not demonstrate significant advantages over the conventional approach in terms of implant survival or functional outcomes. Larger prospective studies are required to confirm these findings.

**Supplementary Information:**

The online version contains supplementary material available at 10.1186/s13018-026-06761-z.

## Introduction

Total knee arthroplasty (TKA) is one of the most frequently performed major orthopedic procedures, with increasing volume driven by population aging and the expansion of indications. The primary goal remains pain reduction and functional recovery, but in recent decades interest has grown in the relationship between alignment accuracy, component positioning, and long-term implant survival [1–4].

The traditional concept of neutral mechanical alignment (± 3°) originated from historical studies suggesting an association with greater implant longevity [2, 4], although subsequent work has downplayed the centrality of this single parameter, emphasizing the multifactorial nature of long-term outcomes (implant design, surgical technique, soft-tissue balancing) [5–7].

There is no evidence demonstrating the superiority of mechanical alignment in terms of clinical or functional outcomes; the available literature rather shows a possible correlation between correct mechanical alignment and implant longevity, but not with improved patient-reported outcome measures [8–11].

Computer-assisted surgery (CAS) was introduced to increase intraoperative precision and reduce inter-operator variability. Navigation has proven to be a useful adjunct, particularly during the early learning curve, improving the accuracy of bone resections and reproducibility of results even for less-experienced surgeons.

Randomized trials and observational studies have demonstrated a reduction in radiographic outliers and greater reproducibility of component positioning with CAS in the short term [5, 6, 11–13].

However, the translation of this improved accuracy into tangible clinical benefits and lower revision rates remains controversial: some series and registry studies have reported advantages in younger patients or specific subgroups [12, 14–17], whereas others, including randomized trials with follow-up beyond 10 years, have found no significant differences compared with conventional techniques [13–16, 18, 19].

In Italy, as in other European contexts, the adoption of CAS has been influenced by economic, organizational, and learning-curve considerations [20–23].

Interest has now shifted toward robot-assisted approaches and patient-specific implantation strategies.

In this study, we report a mean follow-up of 15.4 years comparing conventional and navigated techniques in terms of implant survival (revision for any cause) and clinical–functional outcomes (KSS, WOMAC). This work aims to contribute to the discussion on the true long-term clinical value of CAS, integrating local experience with international evidence.

## Materials and methods

This was a long-term, retrospective comparative analysis. All patients who underwent primary total knee arthroplasty (TKA) between 2005 and 2006 at our Institution were included. All implants were the same cemented, mobile-bearing, ultra-congruent model (SCORE Primary Arthroplasty, Amplitude, Valence, France). Procedures were performed by a single experienced surgeon using either the conventional (CONV) or computer-navigated (NAV) technique.

All TKAs were performed with a ligament-referencing technique through a standard medial parapatellar approach.

The navigated procedures were performed using an imageless computer-assisted navigation system Amplivision (Amplitude, Valence, France). The system relied on intraoperative anatomical landmark registration without preoperative imaging. Optical trackers were fixed to the femur and tibia using percutaneous pins, and real-time feedback was provided to guide bone resections and restoration of mechanical alignment according to a ligament-referencing technique.

The inclusion criteria comprised patients aged 60 years or older at the time of surgery with a diagnosis of primary knee osteoarthritis. Patients with post-traumatic osteoarthritis, rheumatoid arthritis, a history of major knee surgery, or extra-articular deformities requiring corrective osteotomies were excluded. All patients who underwent primary TKA between 2005 and 2006 and met the inclusion criteria were included in the survival analysis. Follow-up status (revision, death, or last available contact) was assessed up to a maximum of 15–16 years after index surgery through clinical records and direct contact when available.

Exclusion criteria included revision total knee arthroplasty, severe systemic conditions that prevented functional assessment, and comorbidities that markedly limited independent ambulation.

All procedures were followed by a standardized postoperative rehabilitation protocol consisting of early active and passive mobilization, full weight bearing with assistive devices from postoperative day one, and structured physiotherapy until the recovery of functional range of motion and an independent gait pattern.

The primary outcome was implant survival, defined as the absence of revision of any prosthetic component for any reason. Revision surgery of any component was considered the event of interest. Patients who died from causes unrelated to the implant or were lost to follow-up were treated as censored observations at the time of death or last available contact, respectively. Mortality unrelated to the implant was recorded for descriptive purposes and as a censoring event, but it was not considered a primary study endpoint.

Secondary outcomes included functional evaluation using the Knee Society Score (KSS) and the Western Ontario and McMaster Universities Osteoarthritis Index (WOMAC), performed in all available patients during in-person clinical assessment. Cases excluded from functional evaluation due to severe comorbidities, loss to follow-up, or non-response were documented.

## Ethical approval

The study protocol was approved by the local Ethics Committee (ICOT, Latina; protocol no. 14/2008). This study was conducted in accordance with the 1964 Helsinki Declaration and its later amendments.

## Statistical analysis

Statistical analysis was performed using SPSS software (IBM Corp., Armonk, NY, USA).

Continuous variables were expressed as mean ± standard deviation (SD) and compared between groups using the Student’s *t*-test for independent samples. Categorical variables were presented as counts and percentages and analyzed using the chi-square test or Fisher’s exact test, as appropriate.

Implant survival was evaluated using Kaplan–Meier survival analysis, considering revision of any prosthetic component as the event of interest. Intergroup differences in survival were assessed using the log-rank test.

A *p*-value < 0.05 was considered statistically significant.

## Results

The initial study cohort comprised 240 consecutive primary TKAs (120 CONV and 120 NAV). During follow-up, 7 patients underwent revision surgery and were counted as events in the survival analysis. A total of 106 patients died from causes unrelated to the implant and were censored at the time of death. Fifty-two patients were lost to follow-up and were censored at the time of their last available contact. At final evaluation, 75 patients were alive and available for clinical–functional assessment (40 CONV and 35 NAV), and only these patients were included in the functional outcome analysis.

The mean age at surgery was comparable between groups, being 78.8 ± 7.2 years (range 60–95) in the CONV group and 79.1 ± 7.4 years (range 60–94) in the NAV group (*p* > 0.05). The sex distribution was similar, with a clear predominance of females (69.2% and 70.0%, respectively). Maximum follow-up was 15–16 years; mean follow-up among patients who reached the final time point was 15.4 years. No statistically significant differences were found in any baseline demographic variable (Table 1).

**Table 1:** Demographic and baseline data of the patients

Variable	CONV (*n* = 120)	NAV (*n* = 120)	*p*-value
Mean age ± SD (years)	78.8 ± 7.2	79.1 ± 7.4	> 0.05
Age range (years)	60–95	60–94	–
Female sex (%)	69.2	70.0	> 0.05
Maximum follow-up (years)	15–16	15–16	> 0.05

At the final follow-up, Kaplan–Meier estimated 15-year implant survival was 70.5% for CONV and 73.4% for NAV (*p* > 0.05), indicating no significant difference in long-term prosthetic endurance between the two surgical techniques. A total of seven revisions were performed throughout the study period: four in the CONV group (two late infections, one acute infection, and one aseptic loosening) and three in the NAV group (one aseptic loosening, one multidirectional instability following a stroke, and one periprosthetic fracture).

Non–implant-related mortality was substantial, reflecting the advanced age and frailty of the population: 49 patients (40.8%) in the CONV group and 57 patients (47.5%) in the NAV group had died from causes unrelated to the implant at the time of final assessment.

At final evaluation, clinical and functional data were available for 40 CONV and 35 NAV patients. All surviving patients underwent direct assessment, either through a clinical visit (23 CONV, 17 NAV) or structured telephone interview (17 CONV, 18 NAV).

Functional outcomes were comparable between the two cohorts. The mean Knee Society Score (KSS) was 170.8 ± 14.2 (range 138–198) for CONV and 172.1 ± 13.7 (range 142–198) for NAV (*p* = 0.46). Similarly, the mean WOMAC score was 20.4 ± 11.9 (range 0–47) in the CONV group and 18.7 ± 10.5 (range 0–44) in the NAV group (*p* = 0.38). No statistically significant differences were observed for either outcome measure, indicating that computer-assisted surgery did not provide measurable clinical or functional advantages in this long-term follow-up population (Table 2).

**Table 2:** Clinical and survival outcomes

Variable	CONV (*n* = 120)	NAV (*n* = 120)	*p*-value
Implant survival (%)	70.5	73.4	> 0.05
Total revisions (n)	4	3	–
Late infection	2	0	–
Acute infection	1	0	–
Aseptic loosening	1	1	–
Multidirectional instability	0	1	–
Periprosthetic fracture	0	1	–
Unrelated deaths (n)	49	57	–
Clinical–functional evaluation (n)	40	35	–
Clinical visit	23	17	–
Telephone interview	17	18	–
Mean KSS ± SD (range)	170.8 ± 14.2 (138–198)	172.1 ± 13.7 (142–198)	0.46
Mean WOMAC ± SD (range)	20.4 ± 11.9 (0–47)	18.7 ± 10.5 (0–44)	0.38

KSS, Knee society score; WOMAC, Western Ontario and McMaster Universities Osteoarthritis Index*

**Fig. 1:**
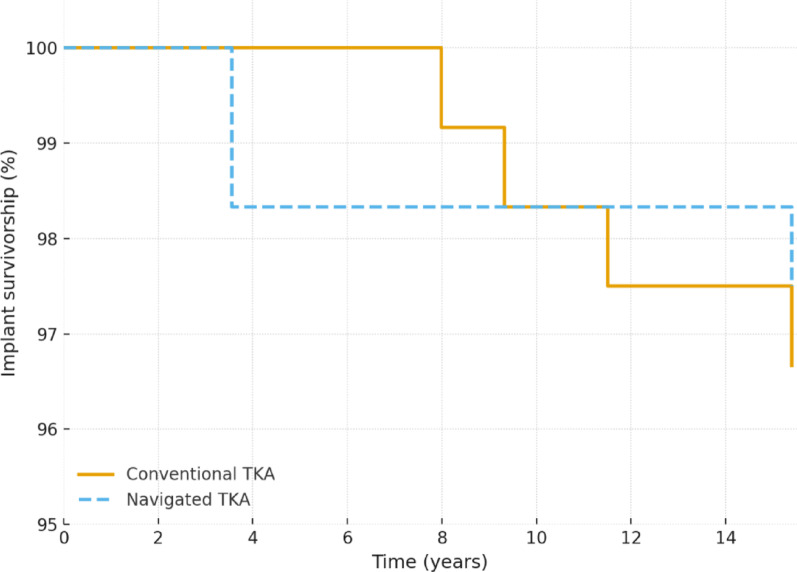
Kaplan–Meier curves illustrating implant survival over 15 years in conventional and navigated TKA; survival patterns are nearly overlapping, with very few revision events and no statistically significant difference between groups

The Kaplan–Meier analysis demonstrated nearly overlapping survival curves for conventional and navigated TKA throughout the 15-year follow-up, with a very low number of revision events and no statistically significant differences between groups (Fig. 1).

## Discussion

 In this long-term comparative study with a mean follow-up of 15.4 years, no significant differences were found between conventional and computer-navigated total knee arthroplasty (TKA) in terms of implant survival or clinical–functional outcomes. Fifteen-year implant survival exceeded 70% in both groups, with very few revision events and nearly overlapping Kaplan–Meier curves. Functional outcomes assessed by Knee Society Score (KSS) and WOMAC were also comparable between techniques.

These findings are consistent with several long-term studies. Parratte et al. reported no association between mechanical alignment and 15-year implant survival, questioning the clinical relevance of small alignment deviations [4]. Similarly, Cip et al. found comparable survivorship between navigated and conventional TKA at 12 years despite better radiographic accuracy in the navigated group [12]. Lützner et al. observed no significant differences in survival or function between techniques at mid- to long-term follow-up [6], and Kim et al., in a randomized trial, failed to demonstrate a long-term clinical advantage of navigation [13]. Registry-based studies, however, suggest that potential benefits of computer-assisted surgery (CAS) may be confined to selected subgroups: de Steiger et al. reported a reduced revision risk with navigation in patients younger than 65 years [16], a population largely underrepresented in the present cohort.

An interesting finding was the absence of periprosthetic joint infection in the navigated group, compared with three infections in the conventional group. Given the very small number of events, this difference is most likely due to chance and the study is clearly underpowered to detect differences in infection rates.

Although computer navigation is often associated with longer operative time and the use of additional fixation pins, which could theoretically increase infection risk, most clinical series have not demonstrated a significantly higher rate of periprosthetic infection with navigated TKA.

 In our experience, navigation may have promoted greater standardization of surgical steps and heightened attention during the learning phase, which could have indirectly contributed to reducing contamination risk. However, this observation should be interpreted cautiously and cannot be considered evidence of a protective effect.

The absence of significant differences in our study may be explained by several factors. First, all procedures were performed by an experienced surgeon, likely reducing inter-operator variability and minimizing the potential advantage of navigation. Second, the use of a single implant model and a standardized ligament-referencing technique limited confounding technical factors. Third, the very low number of revision events reduced the statistical power to detect small differences between techniques.

Moreover, the demographic characteristics of the cohort must be considered. At the time of surgery, TKA was mainly offered to elderly, low-demand patients. Consequently, a large proportion of patients died from causes unrelated to the implant during the long follow-up, and only a small subgroup was available for functional evaluation. This reduced sample size increases the risk of type II error and may mask subtle clinical differences between techniques. This pattern reflects historical indications for TKA and differs from contemporary series that include younger and more active patients.

 Furthermore, the interpretation of patient-reported outcome measures (PROMs) in very elderly patients requires caution. In this population, overall satisfaction may remain high despite relatively modest functional scores, as global mobility and daily activity are often influenced by comorbidities and general frailty rather than knee function alone. In addition, the limited number of patients available for functional evaluation (40 CONV and 35 NAV) further reduces the statistical strength of the PROM analysis.

 The relationship between alignment accuracy and clinical outcomes also deserves consideration. Although navigation has been shown to improve radiographic alignment and reduce outliers [5, 11], multiple studies have failed to demonstrate a direct correlation between small alignment deviations and patient-reported outcomes [8–11]. This supports the concept that long-term success of TKA is multifactorial and depends not only on coronal alignment but also on implant design, fixation method, soft-tissue balancing, and patient-related factors.

This study has several limitations. Its retrospective design and lack of randomization may introduce selection bias. Loss to follow-up and high unrelated mortality reduced the number of patients available for functional assessment. Radiographic alignment was not systematically analyzed, preventing correlation between alignment accuracy and clinical outcomes. Finally, the advanced age of the cohort limits generalizability to younger populations.

From a clinical perspective, these results suggest that, in the hands of an experienced surgeon, conventional and navigated techniques provide comparable long-term outcomes. The choice of technique should therefore consider resource availability, surgical experience, and patient profile. Navigation may still play an important role during the learning curve and in complex cases, and it represents a conceptual step toward robotic and personalized knee arthroplasty, whose long-term benefits remain to be demonstrated.

## Conclusions

At 15 years of follow-up, computer-navigated TKA did not show significant advantages over the conventional technique in terms of implant survival or functional outcomes. Larger, prospective, and multicenter studies are warranted to confirm these results and to further clarify the evolving role of navigation within the context of robotic and personalized knee arthroplasty.

## Supplementary Information

Below is the link to the electronic supplementary material.

Supplementary Material 1.

## Data Availability

The datasets generated and analyzed during the current study are available from the corresponding author on reasonable request.
