# Supplementary material for: Computer-navigated versus conventional total knee arthroplasty: no difference in implant survival at 15-year follow-up
Source: J Orthop Surg Res. 2026 Mar 11;21:266. doi: 10.1186/s13018-026-06761-z (PMC13094171; doi:10.1186/s13018-026-06761-z)
Supplement: Supplementary file 1 — Supplementary Material 1. [file 13018_2026_6761_MOESM1_ESM.docx]

**Ethical Declarations**

**Ethical Approval**The study protocol was approved by the local Ethics Committee (ICOT, Latina; protocol no. 14/2008).

**Consent to Participate**Informed consent was obtained from all patients who were successfully contacted for the study.

**Clinical Trial Number**Not applicable.

**Funding**The authors received no funding for this research.

**Competing Interests**The authors declare that they have no competing interests.

**Availability of Data**The datasets generated and analyzed during the current study are available from the corresponding author on reasonable request.

**Authors' Contributions**Study conception and design: GL, GP
Data collection and analysis: GL, CP
Manuscript drafting: GL
Manuscript revision: AT, SG
